# Smile Renewal: Incisor Restoration Using the Strip Crown Technique

**DOI:** 10.7759/cureus.63716

**Published:** 2024-07-02

**Authors:** Arshjot S Basra, Khyati Manik, Rohan R Khetan, Prem A Sawarbandhe

**Affiliations:** 1 Dentistry, Sharad Pawar Dental College and Hospital, Datta Meghe Institute of Higher Education and Research, Wardha, IND; 2 Conservative Dentistry and Endodontics, Sharad Pawar Dental College and Hospital, Datta Meghe Institute of Higher Education and Research, Wardha, IND

**Keywords:** composite resin, minimal invasion, ellis class ii fractures, facial aesthetics, strip crown technique

## Abstract

Aesthetics has been an important aspect of dentistry along with functionality for decades but its significance is at an all-time high. Hence among techniques that contribute toward this goal, strip crown has shown convincing results. However, its acceptance did not reach its potential, especially among general dentists which could be attributed to its technique-sensitive nature. This case report sheds light on the practicality and reliability of this technique along with various modifications made by authors while striving to improve the quality of treatment provided by the clinician.

## Introduction

Dental trauma is a frequent occurrence, affecting individuals of all ages. Significant tooth fractures or extensive decay can compromise aesthetics, function, and tooth sensitivity in permanent teeth. Aesthetics has been referred to as the fourth dimension of dentistry along with mechanical, physiological, and biological dimensions [1]. Traditional crown placement, while a durable solution, often requires significant tooth reduction and multiple appointments. This case report explores the application of the strip crown technique for restoring a fractured permanent tooth. The strip crown technique, typically used in pediatric dentistry for primary teeth, offers a minimally invasive and possibly single-appointment approach. This method produces a full-coverage, mouth-formed, direct restoration by employing prefabricated thin shells made of celluloid or metal which are filled with tooth-colored composite resin. These shells are placed over the prepared tooth structure, restoring its proper shape and function [2].

The benefits of the strip crown technique in permanent teeth are convincing, such as minimal tooth reduction which translates to a more conservative treatment approach, preserving valuable tooth structure. Strip crowns provide superior aesthetics to other forms of restoration. Additionally, the possibility for single-visit treatment enhances patient convenience and reduces overall chair/treatment time. Given the procedure's popularity, one would anticipate abundant clinical evidence regarding its efficacy. However, the use of strip crowns in permanent teeth has limited documentation compared to their established role in pediatric dentistry. According to a study conducted by Muhamad et al. (2015), only 21.0% of general dentists perform strip crowns, compared to 73.0% of pediatric dentists, indicating a lack of implementation [3].

Recent studies have underlined the efficacy and versatility of the strip crown technique in the management of numerous dental pathologies, including fractures, abrasions, and aesthetic restorations. In a study conducted by Vaghela et al. (2021), the strip crown technique demonstrated comparable clinical outcomes to prefabricated Zirconia crowns in terms of marginal integrity, color match, and patient satisfaction [4]. Furthermore, a recent case report by Muhamad et al. (2015) has acknowledged the lack of published data on the durability and longevity of strip crowns over extended follow-up periods [5].

This case report presents the successful application of the strip crown technique for restoring Ellis class 1 and Ellis class 2 fracture of permanent central incisor in an adult patient. It elaborates on the clinical procedure, discusses the case selection and limitations of this approach in permanent teeth, and explores the potential role of strip crowns as a viable restorative option for selected cases.

## Case presentation

**Figure 1:**
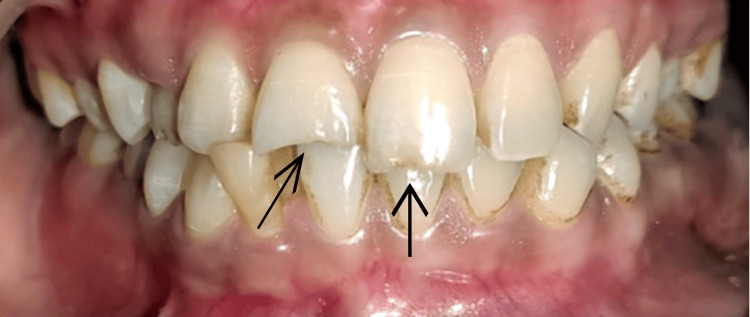
Intraoral View Showing Ellis Class 2 Fracture With Teeth Numbers 11 and 21.

A 27-year-old male patient reported to the Department of Conservative Dentistry and Endodontics, Sharad Pawar Dental College, Wardha, India, with the chief complaint of a fractured tooth in the upper front region of the jaw and wanted to get it restored. The patient had a history of road traffic accident one year back, which led to a fracture in the upper front region of the jaw; there was no associated history of mobility, pain, or swelling. No significant medical or dental history was present. On extraoral examination, the face was bilaterally symmetrical, lips were competent, and on the temporomandibular joint (TMJ) examination, it was bilaterally smooth and synchronous with no clicking. On intraoral examination, Ellis class 2 fractures with teeth numbers 12 and 21 were present. However, tooth structure loss with tooth number 11 was present on both the buccal and palatal aspects, whereas with tooth number 21, tooth structure loss was majorly seen in the palatal aspect (Figure 1).

**Figure 2:**
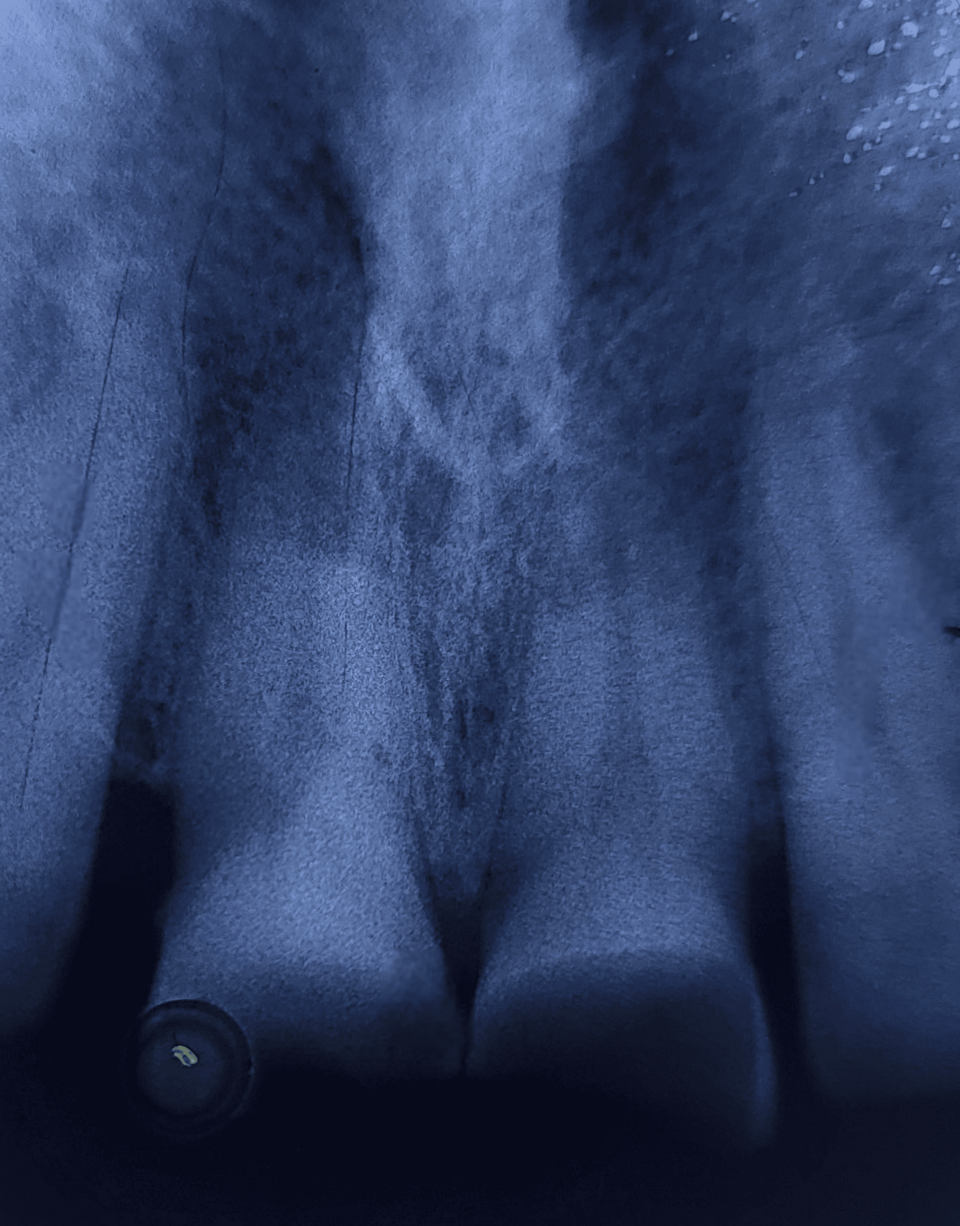
Intraoral Periapical Radiograph (IOPA) Showing Ellis Class 2 Fracture With No PDL Widening or Periapical Radiolucency. Ellis class 2 fracture with teeth numbers 11 and 21 is shown along with coarse alveolar bone trabeculae which might give a blurry appearance on an IOPA. No PDL widening is visible. PDL: periodontal ligament

The neural sensibility test was performed by heat and cold test along with electric pulp testing (Walden Electric Pulp Tester, Walden Dental Products, New Delhi, India), heat test was performed using a hot ball burnisher while the cold test was performed by using endo ice (Coltene Vitality Control Endo-Frost, Coltene Whaledent, Cuyahoga Falls, USA). While performing electric pulp testing with tooth number 11, the control tooth taken was 12, and readings were 14 μA for the former and 25 μA for the latter. Similarly, while performing the test for tooth number 21, the control used was 22, and readings were 12 μA for the former and 25 μA for the latter. To form a diagnosis, an intraoral periapical radiograph (IOPA) was taken with teeth numbers 11 and 21. On the radiograph, radiolucency involving the incisal edge of teeth numbers 11 and 21 was observed; radiolucency did not involve the pulp chamber, along with no visible periodontal ligament (PDL) space widening and no periapical radiolucency (Figure 2).

On the basis of a pulp neural sensitivity test, clinical examination, and radiographic examination, the case was diagnosed as asymptomatic reversible pulpitis with teeth numbers 11 and 21.

Treatment

Once the procedure was explained to the patient, rubber dam isolation was done for teeth numbers 12, 11, 21, and 22. This was followed by shade matching of the composite material, which was done by placing the composite buttons on the unprepared tooth surface and polymerizing it before clinically observing the best shade for restoration (Video 1).

**Video 1:** Shade Matching of Resin Composite

Once the shade had been selected (A2 composite Dentsply Spectrum® Universal Micro Hybrid Composite, Germany), beveling of the tooth surface was done using TF-12 bur (Mani® Diamond Bur, Utsunomiya, Japan) (Video 2).

**Video 2:** Beveling of the Tooth Surface

**Figure 3:**
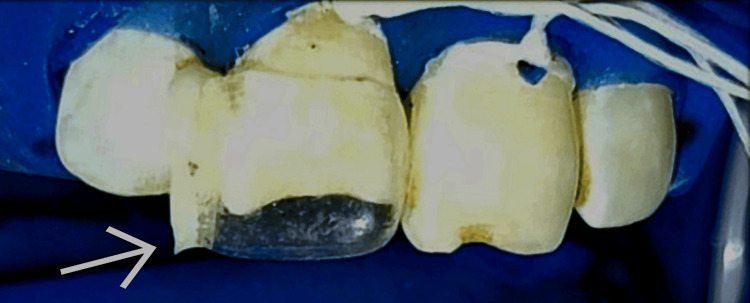
Trial Check of Strip Crown Form Against the Tooth Surface.

Strip crown selection was done by placing the incisal edge of celluloid shells against tooth number 11 to confirm the correct size. A prefabricated maxillary central incisor strip crown (Tor Vm Anterior Transparent Crown; Tor Vm, Moscow, Russia) was selected, and its dimensions were coordinated according to the tooth size. This was followed by piercing the crown with a sharp explorer at the distal incisal angle so as to create a vent for escape of air bubbles that might get entrapped in the crown while filling the crown with composite later. Trimming of the selected crown at the gingival margin using a curved scissor was done so as to do a trial check of crown form by placing it against the tooth surface (Figure 3).

**Figure 4:**
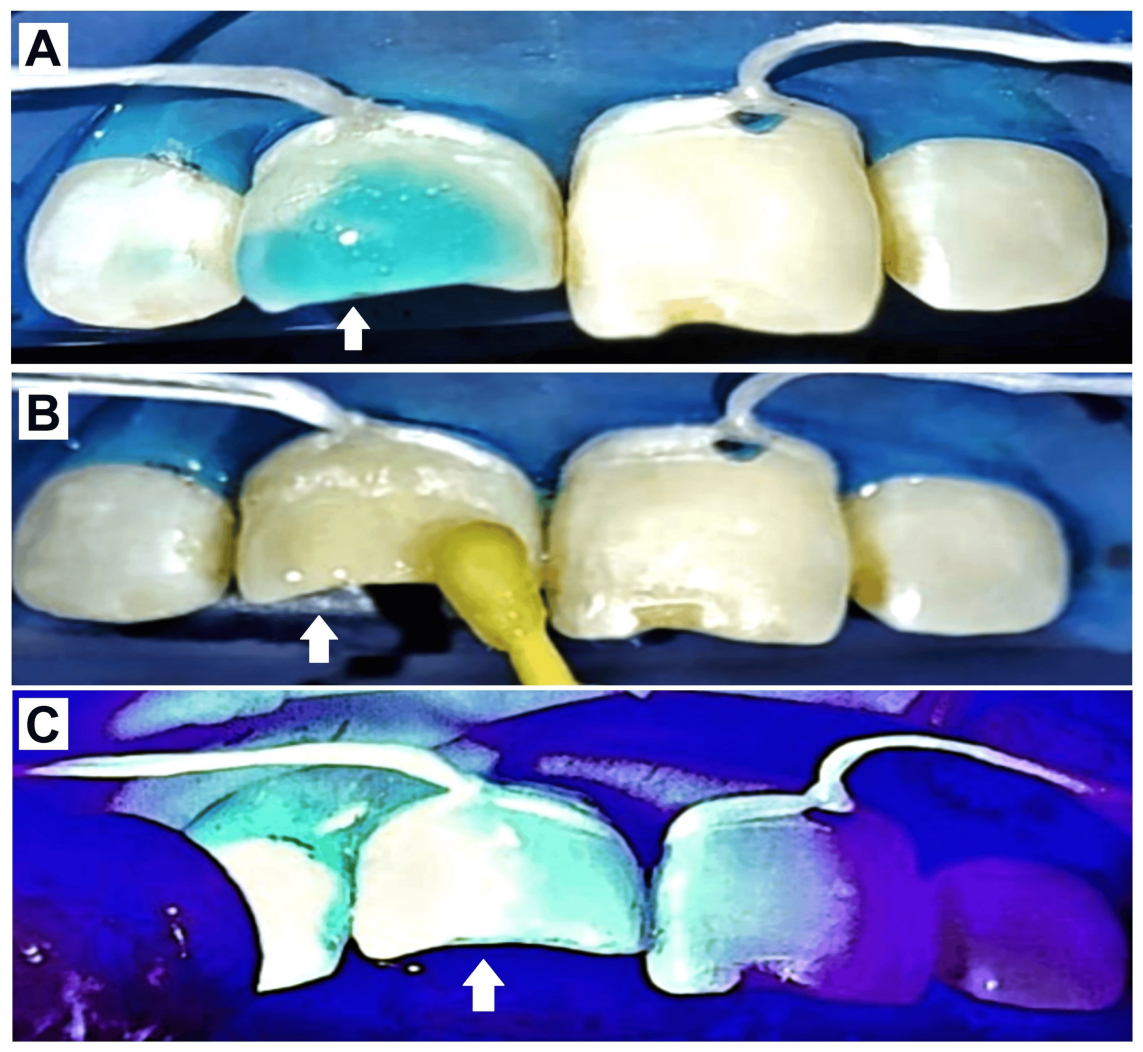
Clinical Steps in Composite Restoration (A) Etching with 37% phosphoric acid; (B) Bonding agent application by applicator tip; (C) Light curing of applied bonding agent.

Etching using 37% phosphoric acid (Prime Dental Products Pvt Ltd., India) was done for 15 seconds, followed by rinsing the tooth with water and air drying with the help of a three-way syringe. Later bonding agent (3M ESPE Adper Single Bond 2; 3M ESPE, India) application onto the prepared tooth surface was done by applicator tip in two layers which were later light cured (Woodpecker O Light Cure Unit; Woodpecker Dental Co., Ltd., Guilin, China) for 20 seconds (Figure 4).

Composite material was filled in the crow form, which was pressed onto the lingual and buccal tooth surface of tooth number 11 by gentle pressure. This caused the excess composite to flow out of the strip crown (Video 3).

**Video 3:** Composite Filled Strip Crown Being Pressed Onto the Lingual and Buccal Tooth Surface by Gentle Pressure.

**Figure 5:**
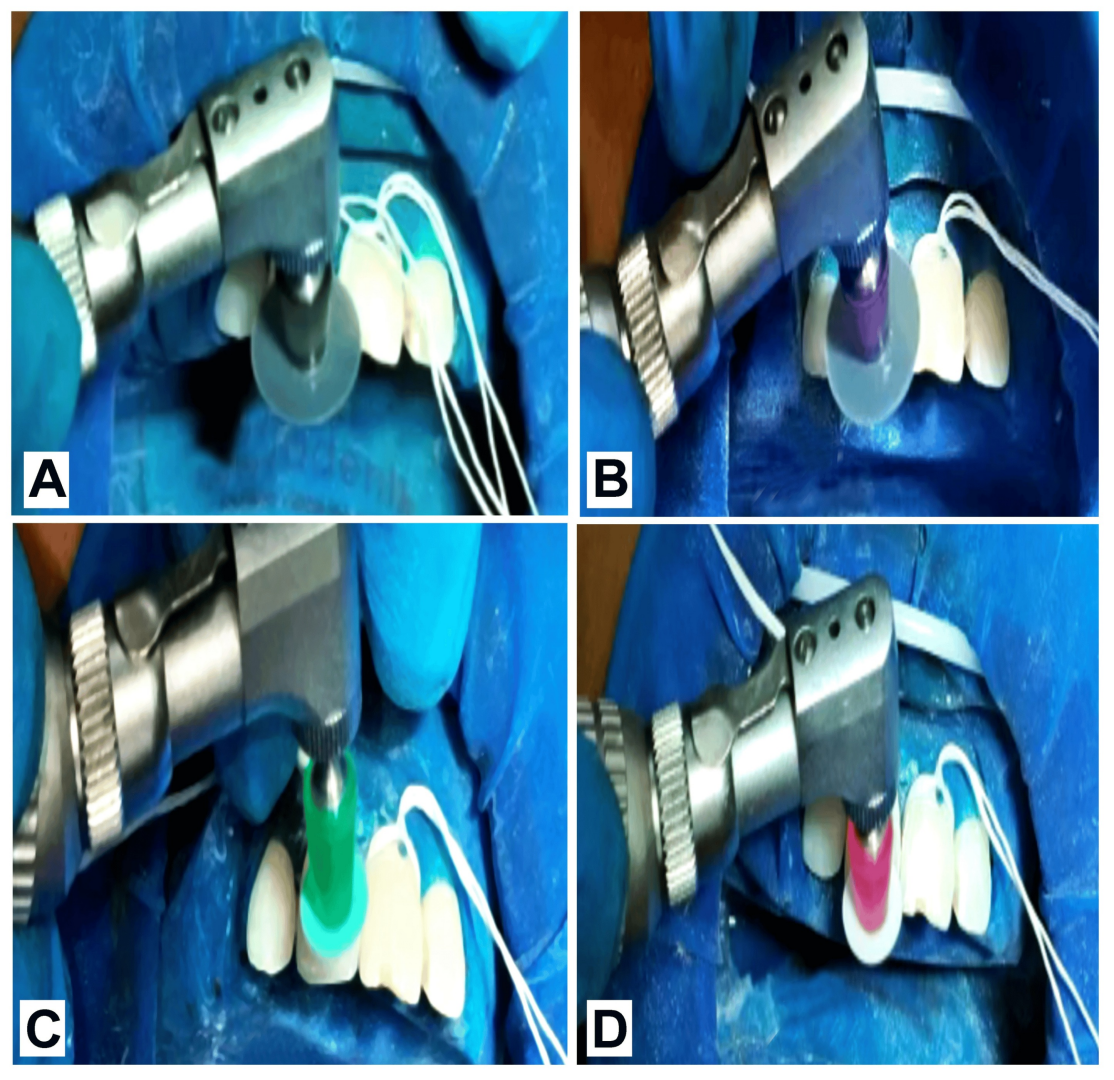
Polishing With Series of Abrasive Disks (A) Coarse disk for contouring; (B) Medium disk for finishing; (C) Fine disk for polishing; (D) Super fine disk for super polishing.

After that, the crown was light-cured for 20 seconds. To facilitate easy separation, the crown was cut from the distal aspect and separated, once the composite resin had fully set. For finishing and polishing, extra fine tapered diamond TF-11 bur (Mani® Diamond Bur, Utsunomiya, Japan) was employed for coarse finishing the composite. Final finishing and polishing were done with a series of abrasive disks (Shofu Super Snap Mini Kit, Kyoto, Japan) (Figure 5).

**Figure 6:**
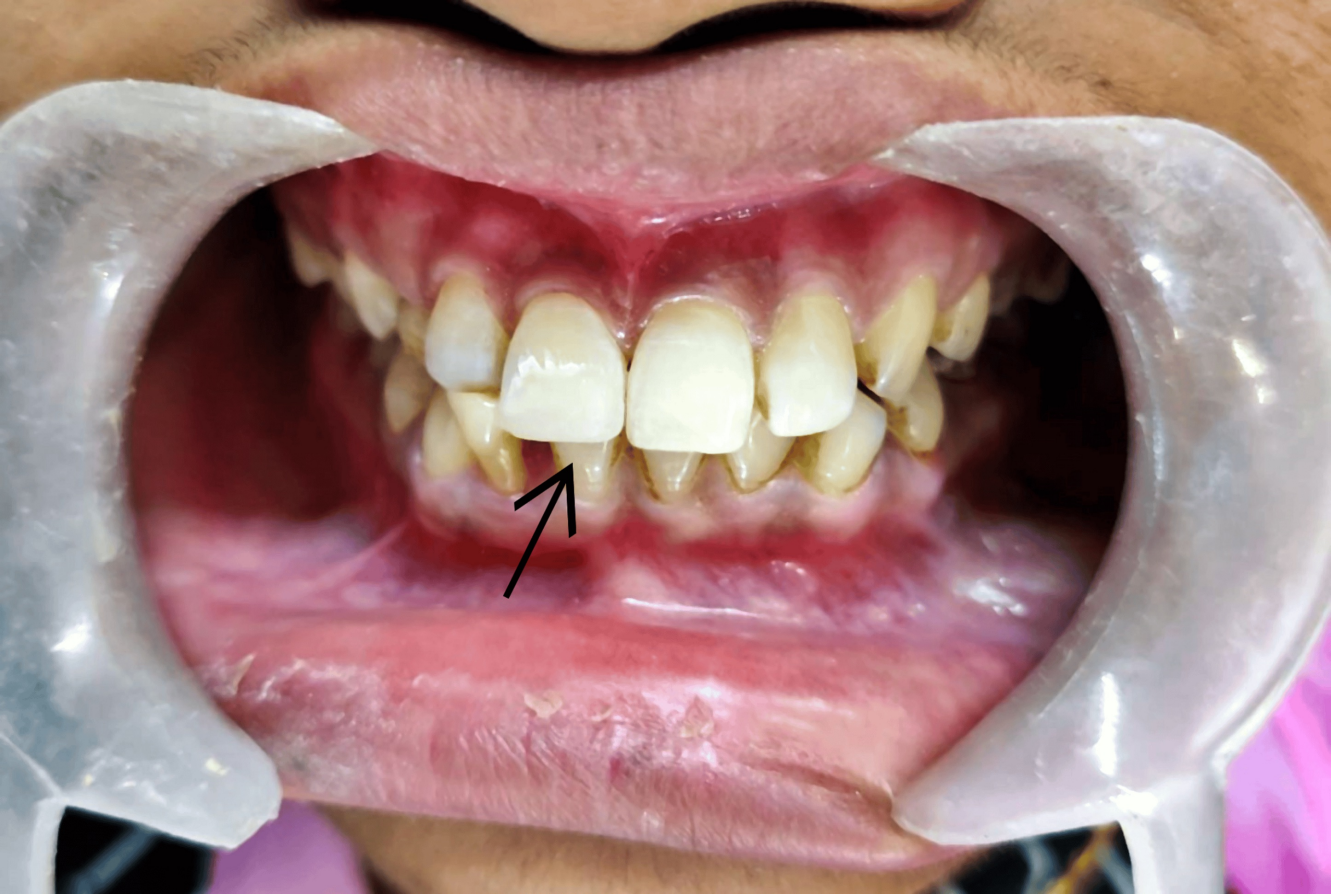
Postoperative Photograph of the Patient's Dentition

The adjacent tooth, tooth number 21, was restored by free-hand composite technique as enamel margins were mostly still intact buccally. Postoperatively, the fracture line was no longer visible, and the patient was satisfied with the results (Figure 6).

## Discussion

The strip crown technique has emerged as a popular choice among pediatric dentists for restoring primary anterior teeth. This technique offers several advantages, particularly in young patients, but its successful application relies on understanding its ideal uses and limitations. In one such clinical study that focused on direct composite full-coverage crowns, it was revealed that, overall, 89% of cases restored by the mentioned technique had cervical discoloration as a result of chip fractures, which resulted in microfiltration and ultimately loss of adaptation [6]. Another study compared the strip crowns technique with Zirconia crowns based on various variables like gingival index, crown retention, color match, opposing tooth wear, recurrent caries, crown contour, and marginal integrity. In which Zirconia crowns showed a 100.0% success rate whereas, among all the samples of strip crowns 3.60% showed slight shade mismatch at three months, and 29.40% showed color mismatch at nine months follow-up. The reason for color mismatch could be microleakage due to chipping/loss of material, incomplete light curing of resin composite, or blood contamination that may occur during the process and change the resin composite shade [4]. In a study conducted in the 1990s, a 100% retention rate with the composite strip crown technique was reported in a sample size of 92 teeth with a one-year follow-up [7]. Mentioning another such study by Kupietzky et al. (2003) in which clinical and radiological findings from 112 composite resin strip crowns in 40 children were documented, the crowns were found to have an 88.0% retention rate on a follow-up period of 18 months [8]. One year later, parental satisfaction with the aesthetic appearance of the strip crowns was evaluated using the same retrospective study sample in which 78.0% of parents reported being "very satisfied" with the crowns, and durability was significantly correlated with their level of satisfaction [8,9].

Various authors have explored modifications to the strip crown technique. In places lacking enamel, such as the gingival edges, authors have tried dentin replacement with a resin-modified glass ionomer cement (RMGIC) to prevent composite materials from debonding by placing a layer of RMGIC to protect all exposed dentin prior to the seating of the crown form filled with composite resin [10]. Another modification that was explored so as to increase the longevity and retention of the strip crown is the composite resin short post, or "mushroom undercut," in which an undercut is made into the dentin to aid in the retention of the crown. This study assessed 243 patients' clinical outcomes retrospectively using the short post method on 625 composite resin strip crowns. It was discovered that composite resin strip crowns showed satisfactory retention to last till normal exfoliation of the primary tooth with the right case selection, mechanical short post design, and crown-to-root ratio [11]. Hence, strip crowns offer a valuable tool for restoring anterior teeth, but judicious use is key to ensure long-term success. Judicious use of strip crowns involves meticulous case selection and optimizing treatment planning. Understanding of indication and contraindication is hence essential in such cases.

Indications for the strip crown technique

(A) Fractured Incisors

The primary function of the strip crown technique is to restore incisors with significant coronal tooth structure loss due to fractures or trauma. These fractures can be caused by falls, accidents, or even biting on hard objects. Strip crowns efficiently enclose the remaining tooth structure, providing structural support and avoiding further damage from masticatory forces or oral habits [5].

(B) Extensive Caries

In cases where deep caries have compromised primary incisors, but adequate tooth structure remains, strip crowns offer a feasible restoration option. The technique allows for the removal of decayed tooth structure followed by the placement of the composite crown, effectively halting the progression of caries and restoring functionality [12].

(C) Hypomineralization

In incisors with enamel defects or hypomineralization that impact aesthetics and function, strip crowns can provide a substantial benefit. Hypomineralization can lead to weakened enamel, discoloration, and sensitivity. Strip crowns not only address these cosmetic concerns by creating an aesthetic restoration but also protect the remaining tooth structure from further damage or discomfort [5].

Contraindications for the strip crown technique

(A) Insufficient Tooth Structure

Even though minimally invasive, the strip crown technique still requires a minimum amount of sound tooth structure for proper adhesion and retention of the composite. If the remaining tooth structure is insufficient after caries removal or due to fracture, alternative restoration methods, such as stainless steel crowns, might be required to ensure crown stability [12].

(B) Deep Overbites

In cases with a deep overbite, where the maxillary incisors significantly overlap the mandibular incisors, the increased occlusal stress placed on the restored tooth can lead to crown fracture or dislodgement of the restoration. Clinicians might go for alternative restorations, which are designed to withstand high occlusal forces associated with cases of deep overbites [5].

(C) Periodontal Disease

If the primary tooth suffers from periodontal disease affecting the supporting bone structure, a strip crown might not be a viable option. Periodontal disease can compromise the stability of the tooth, affecting the longevity of restoration. In such cases, alternative restoration or potential tooth extraction might be necessary [12].

## Conclusions

The strip crown technique offers a valuable means for clinicians to restore primary as well as permanent anterior teeth. While there is still a lack of documentation about its use in adult patients, understanding the indications and contraindications for its use is crucial for achieving successful and long-lasting treatment outcomes, which enables its use in permanent dentition as well. The minimally invasive nature, efficiency, and aesthetic results of the strip crown technique make it a favorable option for a variety of situations involving fractured, carious, or hypomineralized primary incisors in young patients.
